# Development of High-Pressure Extraction and Automatic Steam Distillation Methods for *Aronia mitschurinii*, Juvenile Ginger, and Holy Basil Plants

**DOI:** 10.3390/molecules30102199

**Published:** 2025-05-17

**Authors:** Sara Lahoff, Ezra E. Cable, Ryan Buzzetto-More, Victoria V. Volkis

**Affiliations:** Department of Natural Sciences, The University of Maryland Eastern Shore, Princess Anne, MD 21853, USA; selahoff@umes.edu (S.L.); eecable1@umes.edu (E.E.C.); rrmore@umes.edu (R.B.-M.)

**Keywords:** high-pressure extraction, automatic steam distillation, extraction methods, *Aronia mitschurinii*, holy basil, juvenile ginger, polar solvents, nonpolar solvents

## Abstract

Sample preparation is the most time-consuming part of phytochemical, agricultural chemical, and food science studies and is constantly being improved. This includes the development of modern extraction methods, such as high-pressure extraction and automatic steam distillation. These methods feature high reproducibility, low time consumption, and the ability to run several parallel samples. However, the ideal parameters for processing plant materials using these methods have not been fully explored. These parameters include those that produce the highest yield and those that produce yields comparable to less modern extraction techniques, which would allow for a comparison of data to a wide range of preexisting data obtained from plant materials in different growing locations and climates. As such, this study examined extracts produced by reflux extraction, high-pressure extraction, and traditional and automatic steam distillation for three plants: aronia, holy basil, and juvenile ginger. High-pressure extraction methods were developed to produce extracts similar to those produced by reflux extraction, while automatic distillation methods were developed to produce high essential oil yields. The automatic steam distillation yields were 55.81 ± 1.97 mg/g of holy basil, 61.52 ± 0.61 mg/g of ginger, and 45.79 ± 1.38 mg/g of aronia. The high-pressure extraction yields were 11.09 ± 1.46 mg GAE/g of holy basil, 154.50 ± 17.10 mg of anthocyanins/mL of aronia, 6.60 ± 0.55 mg GAE/g of ginger, and 3.27 ± 0.25 mg GAE/g of ginger. These were compared to reflux yields of 32.71 ± 5.22 mg GAE/g of holy basil, 253.00 ± 39.56 mg of anthocyanin/mL of aronia, and 3.34 ± 2.07 mg GAE/g of ginger.

## 1. Introduction

Sample preparation is an essential part of chemical projects; it has proven to be particularly time-consuming and expensive when it comes to working with plant materials, accounting for 75% of the project time. It can raise a project’s cost not only because it is time-consuming but also because it requires large amounts of energy and materials and produces potentially hazardous waste [1]. As such, there have been various innovations to minimize the time and cost of sample preparation to focus more on data collection. These innovations have recently included high-pressure extractors and steam automatic distillers. However, even though these modern methods have gained widespread use in some chemical fields, such as in the pharmaceutical chemistry field, they have yet to gain any notable use in other fields, such as the agricultural and phytochemistry fields.

This study aims to use these instruments to develop quicker methods for essential oil and phytochemical extraction from three crops: juvenile ginger, holy basil, and aronia.

**Figure 1:**
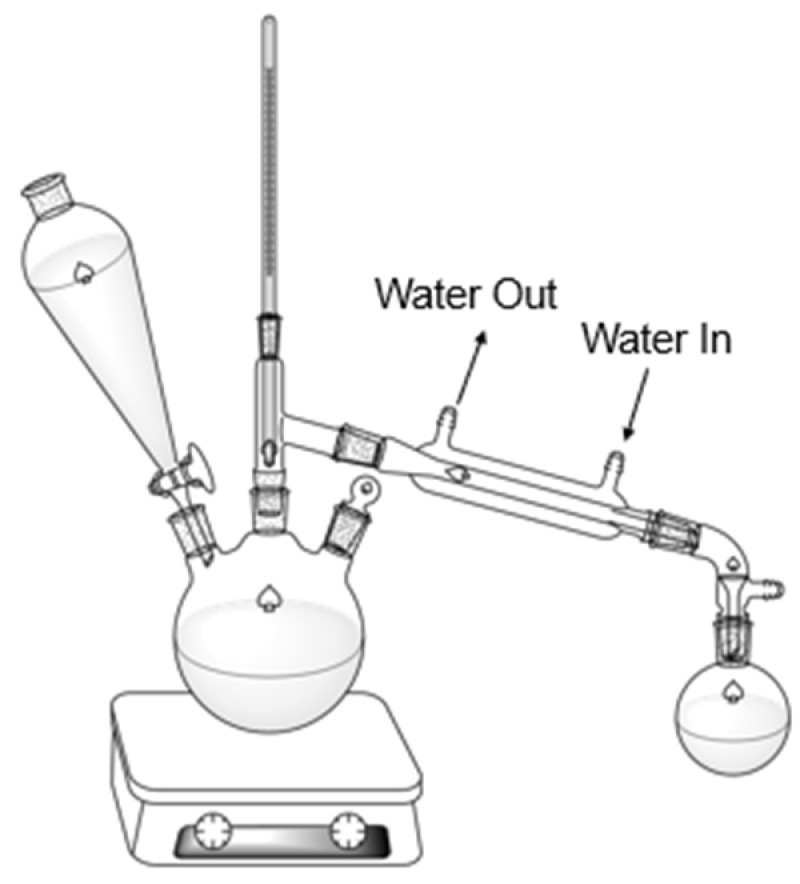
Direct steam distillation setup.

Steam distillation is a distillation process commonly used to extract essential oils from plant materials. Through this method, steam is generated from a source and is injected into plant materials, carrying essential oils, and it is then passed through a condenser and collected in a separate container, as shown in the direct steam setup in Figure 1 [2].

This method is commonly used for the extraction of essential oils from plants such as ginger due to it being less costly to operate than other methods while being environmentally clean due to the only solvent being water [3].

However, this method takes an extremely long time to conduct, taking days on end; requires ample amounts of water; risks sample deterioration due to long exposure to heat sources; and produces low yields. As such, there have been many innovations to improve upon standard steam dilation, such as microwave steam distillation and steam distillation–solvent extraction [2]. Automatic distillation, one of these successors, serves as a way to shorten that time while providing a higher yield by enabling more control over the distillation process.

**Figure 2:**
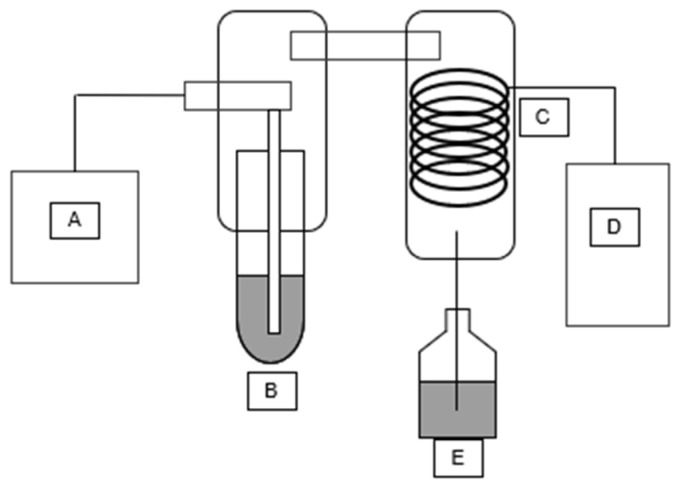
Automatic steam distillation setup: steam source (A), injected into a sample (B), and then passed through a condenser coil (C) cooled by a water chiller (D) into a collection vial (E).

Automatic steam distillers work similarly to regular methods of steam distillation. As such, steam is generated from a steam source (A), injected into a sample (B), and then passed through a condenser coil (C) cooled by a water chiller (D) into a collection vial (E) (Figure 2).

However, automating the steam distillation process allows for the easier reproducibility of results. This is achieved by automating the parameters of the distillation process, such as temperature, steam intensity, and distillation time, causing them to be more consistent across different trials. Additionally, it may speed up the distillation process by enabling intensified steam power and an improved condenser coil design [4].

Despite the more intense steam power and shorter steam times, automatic steam distillation has been shown to yield results comparable to those of more traditional methods. For instance, a study was performed comparing automatic steam distillation to regular steam distillation regarding determining the alcohol strength of spirit drinks. This study found that the results produced by an automatic steam distiller correlated well with those produced by regular steam distillation. The study ultimately found that automatic steam distillation provided a faster, more cost-effective method for the steam distillation of alcohol with fewer sources of error [5]. This same lab conducted a study on more recent automatic steam distillers and found that the reproducibility and reliability of results have continued to improve as automatic steam distillers have been innovated [4].

Beyond its use in alcohol extraction, automatic steam distillation has also gained use in essential oil extraction. This is in part due to steam distillation already being widely used for essential oil extraction due to it producing oils in a simplistic and low-cost manner. This has been seen in the increasing number of patents for steam distillation technology for essential oil distillation [3]. However, automating this process is necessary to improve yield due to the volatility of essential oils and how they may easily be destroyed or modified by the parameters of the distillation process, such as temperature and distillation time [6].

**Figure 3:**
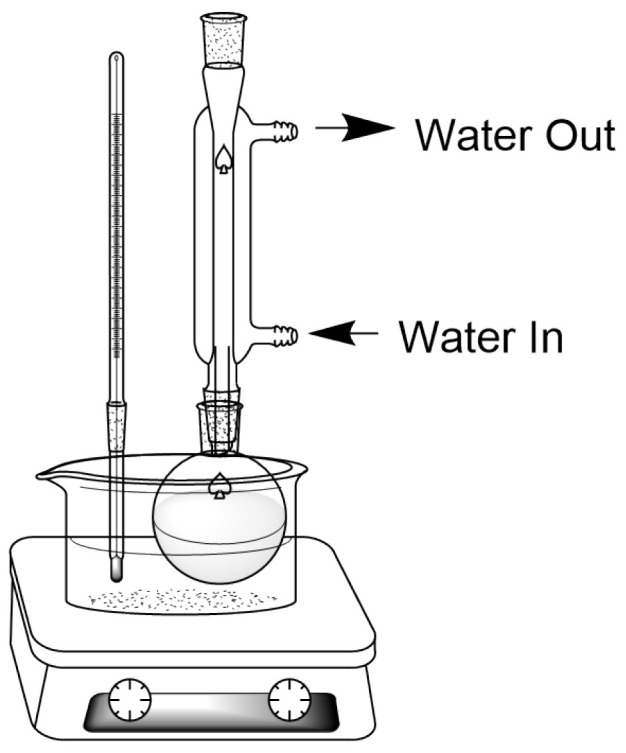
Standard reflux apparatus setup.

While there are a wide variety of different types of extraction, high-pressure extraction is generally used in place of solid–liquid extraction. In particular, our previous research utilized reflux extraction (Figure 3) to obtain extracts from plants for phytochemical screening.

For instance, our previous research utilized reflux extractions of ginger in 50% ethanol in water and in methanol. The extracts produced were used for phytochemical screening to measure the total flavonoids, total polyphenols, and tannins, as well as qualitatively observe the presence of alkaloids, flavonoids, tannins, saponins, glycosides, steroids, anthraquinones, phenols, and oxalates [7].

However, solid–liquid extraction has a variety of drawbacks, including high solvent consumption, a long extraction time, and the risk of solute degradation due to long exposure to high temperatures. As such, there have been a variety of different successors to solid–liquid extraction, including microwave-assisted extraction and supercritical fluid extraction [8].

In contrast to regular extraction methods, for which the time required can range from a few hours to multiple days, high-pressure extraction may speed up the process considerably. This is due to high-pressure extractors subjecting the extract and solvents to a high pressure, lowering the boiling point of the solvents used, and allowing the extraction to take place using higher temperatures than regular extraction. This aids in the desorption of molecule–molecule interactions and provides molecules with more energy, increasing the likelihood of overcoming the existing activation barrier. It also aids in improving the diffusion rate and speeding up the extraction. As such, they allow for a faster extraction process while also minimizing solvent loss by minimizing evaporation [9,10,11]. The type of solvent or their mixtures, the amount of solvent and substrate, the pressure, and the temperature can be regulated to obtain the optimal extraction conditions.

**Figure 4:**
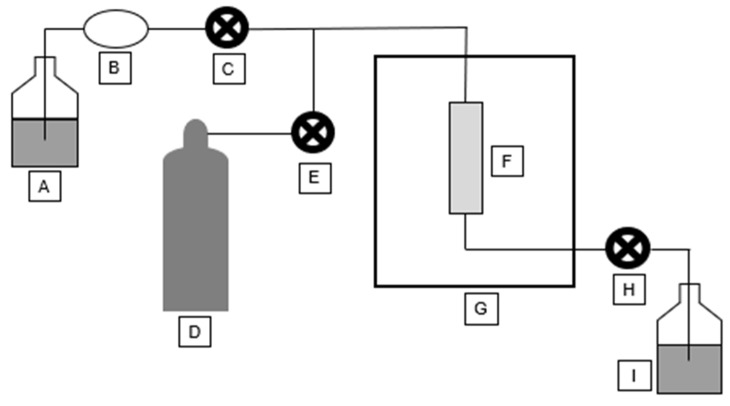
High-pressure extractor schematic.

A schematic of a high-pressure extractor can be seen in Figure 4. Solvent is taken from a solvent container (A) by a pump (B) through a pump valve (C), and it is passed into an extraction cell (F) heated by an oven (G). The solvent is then pressurized using nitrogen gas (D) supplied to the instrument through a purge valve (E). After a period of time, the extract is then flushed through a static valve (H) into a collection vial (I). Nitrogen gas is also used to flush any remaining extract.

Promising results from high-pressure extraction have been widely reported. For instance, a study analyzed the use of high-pressure extraction to extract antioxidants from the hulls of djulis (*Chenopodium formosanum*), a cereal plant found in Taiwan. This study extracted antioxidants such as flavonoids and gallic acids under pressures ranging from 100 to 600 MPa and compared the resulting yields to those produced using conventional oscillation extraction. The study found that all yields produced by the high-pressure extractor were higher than those produced by conventional oscillation extraction. Additionally, the yields increased with pressure, with 600 MPa producing the highest yields [12].

Additionally, there was a study that examined the use of high-pressure extraction on tomato (*Solanum lycopericum*) pulp due to its high antioxidant content. In particular, this study examined the optimal way to obtain the highest extraction yield, flavonoid content, and lycopene content. This study found that high-pressure extraction produced an increased antioxidant capacity, flavonoid content, and lycopene content when compared to conventional extraction [13].

While these studies found that high-pressure extraction produced higher yields than regular extraction, they often only focused on one of a few parameters that may be changed using a high-pressure extractor. As such, this research focuses on examining each parameter of a high-pressure extractor to develop more complete methods.

It is important to point out that, while the goal of developing new instrumental methods, especially for food technology, is often to maximize the yield, the goal of phytochemical studies typically includes comparing the phytochemical content of plant material to preexisting data obtained under different growing conditions, such as different growing locations. Much of these preexisting data were obtained using more traditional forms of extraction, such as reflux extraction. As such, it is necessary to develop methods that not only optimize extraction yields but also produce similar yields to more traditional forms of extraction. This would allow for a better comparison of the effects of other factors, such as climate conditions, irrigation, areas of growth, fertilizers, and pesticides, on phytochemical content by minimizing the influence of different extraction techniques on extraction yield. As such, high-pressure extraction methods were developed not only to optimize phytochemical yield but also to yield comparable results to reflux extraction. In this study, individualized high-pressure extraction methods were developed for three crops: juvenile ginger, holy basil, and aronia.

Ginger is commonly used as a spice and has been shown to have a variety of positive impacts on human health. These impacts are due to its high content of beneficial nutrients and bioactive compounds, causing it to have anti-inflammatory, antioxidant, anti-nausea, anti-diabetic, and anti-cancer properties. Some of these compounds include 6-gingerol, polyphenols, and flavonoids [14].

Our research focused on juvenile ginger due to its potential to have more potent health benefits while being easier to grow and process [15]. For instance, our previous research showed that ginger grown for 9–11 weeks has the highest concentration of phenolic compounds, greater amounts of antioxidant activity, and more potent anti-obesity potential [7].

The key part of ginger used is its rhizome. As such, it is also used in this research as an example of processing similar plants also used for their rhizomes. These include plants that have been shown to contain beneficial compounds, such as other members of the genus *Zingiber*, including *Z. montanum*, *Z. cassumunar*, and *Z. corallinum*, which have been shown to contain various amounts of terpenes and essential oils [16].

Holy basil (*Ocimum sanctum*), or Tulsi, is widely used in Ayurvedic medicine. Tulsi is indigenous to Asia, Africa, and Central and South America, and it is most commonly identified as either *Rama Tulsi* or *Krishna Tulsi* [17]. The crop is known for having valuable components such as eucalyptol, camphor, and eugenol, which contribute to the plant providing fever relief, being antimicrobial, and treating a wide variety of illnesses [18]. Other valuable phytochemicals found in the crop include tannins, polyphenols, and flavonoids, all of which contribute to the plant’s anti-inflammatory properties.

While there are many herbs across the world, not many crops are comparable to holy basil. Holy basil stands out due to its unique phytochemical composition and high yields of essential oils. One similar crop is known as sweet basil (*Ocimum basilicum*). When comparing sweet basil to holy basil, both were shown to have a high essential oil content; however, holy basil was shown to have higher concentrations of essential oils than sweet basil after both steam distillation and hydrodistillation [19].

*Aronia mitschurinii*, referred to as “Aronia” in this paper, is a variety of the genus *Aronia*. While the three most common species of *Aronia*, namely, *A. arbutifolia*, *A. melanocarpa*, and *A. prunifolia*, are native to North America, this type of *Aronia* can be traced back to 20th-century Russia, where the North American varieties of *Aronia* were hybridized with *Sorbus aucuparia* and other members of the Pyrinae. In particular, studies have shown that *Aronia mitschurinii* is the product of ×*S. fallax* (*A. melanocarpa* × *S. aucuparia*) backcrossed with other *Aronia* species. However, *Aronia mitschurinii* remains genetically similar to the other three types of *Aronia* [20].

*A. mitschurinii* contains four main classes of phenolics: polyphenols, tannins, flavonoids, and anthocyanins. Polyphenols, characterized by multiple phenolic rings, include gallic acid. The primary polyphenol classes are phenolic acids and flavonoids. Flavonoids, distinguished by a three-ring structure, include quercetin. Their major subgroups are flavones, flavanones, flavanols, isoflavones, anthocyanidins, and anthocyanins. Anthocyanins, a flavonoid subtype, resemble anthocyanidins but have a glycosidic linkage. The key anthocyanins in *A. mitschurinii* are cyanidin-3-galactoside (Cy3Gal) and cyanidin-3-glucoside (Cy3Glu). *Aronia mitschurinii* is known to contain a higher concentration of anthocyanins than other fruits [21].

The harvesting of *Aronia mitschurinii* is important for maintaining a high concentration of phytochemicals. The concentrations of polyphenols, flavonoids, anthocyanins, and proanthocyanins vary during the crop’s harvesting season. While the fruit appears to be fully ripened, changes occur in the phytochemical content of the fruit during the roughly 1-month period when it is harvestable. Harvesting the fruit during times when there is an increased concentration of antioxidants is useful for creating more potent extracts [22].

In this study, we utilized high-pressure extraction and automatic steam distillation to develop methods for producing extracts with concentrations of phytochemicals comparable to those produced by traditional methods for the three plants mentioned above, covering various types of harvests, namely, berries, rhizomes, and medical herbs. In this study, the results of traditional extraction and wet distillation were compared with results obtained under different sets of parameters for high-pressure extraction and automatic wet distillation. The main aim was to find parameters that produce results similar to traditional sample preparation rather than maximizing the yield. The reason for this was to be able to compare data published over almost a century for different types of crops, across many areas of growth, and with different cultural management practices applied.

## 2. Results and Discussion

### 2.1. Distillation Results

The distillation results were varied to determine which parameters result in the highest yields. Steam time was the first parameter tested, followed by % steam power.

**Figure 5:**
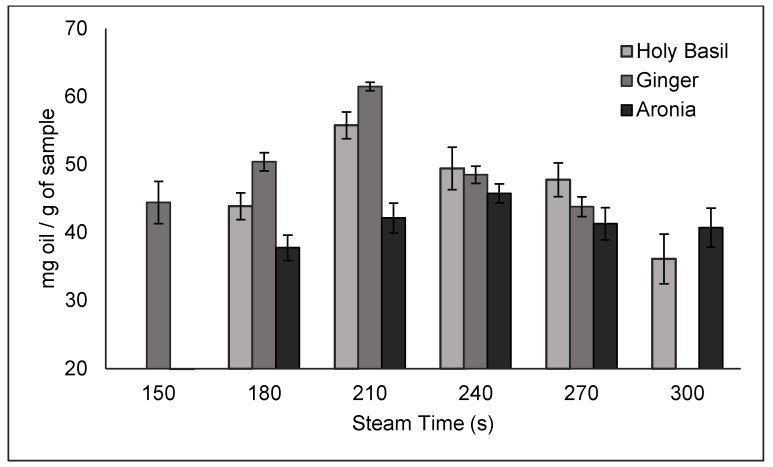
Essential oil yields across different steam times.

The results of the time trials are shown in Figure 5. Based on these data, 210 s was determined to be the optimal steam time for ginger and basil, resulting in a yield of 61.52 ± 0.61 mg/g of ginger and 55.81 ± 1.97 mg/g of basil. Meanwhile, 240 s was the optimal steam time for aronia, with a yield of 45.79 ± 1.38 mg/g of aronia. For all trials, 90% steam power was used due to it being recommended by the manufacturer.

**Figure 6:**
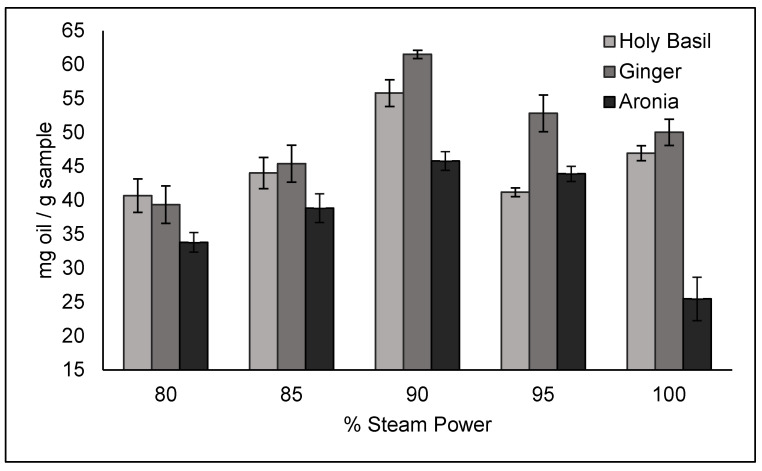
Essential oil yields for different % steam power values.

The results of the percentage steam power trials are presented in Figure 6. A steam time of 210 s was used for ginger and basil, while a steam time of 240 s was used for aronia, due to them being the optimal times determined by the time trials. A steam power of 90% was found to be the optimal steam power for all three crops, resulting in a yield of 61.52 ± 0.61 mg/g of sample for ginger, 55.81 ± 1.97 mg/g of sample for basil, and 45.79 ± 1.38 mg/g of sample for aronia. A comparison of the ginger and basil results obtained under optimal conditions to other literature values can be seen in Table 1.

### 2.2. Pressure Extraction Results

Shown below are the polyphenol and anthocyanin results for the pressure and temperature trials. The goal was to obtain results as close as possible to those produced by reflux extraction.

#### 2.2.1. Polyphenols of Ginger and Holy Basil

**Figure 7:**
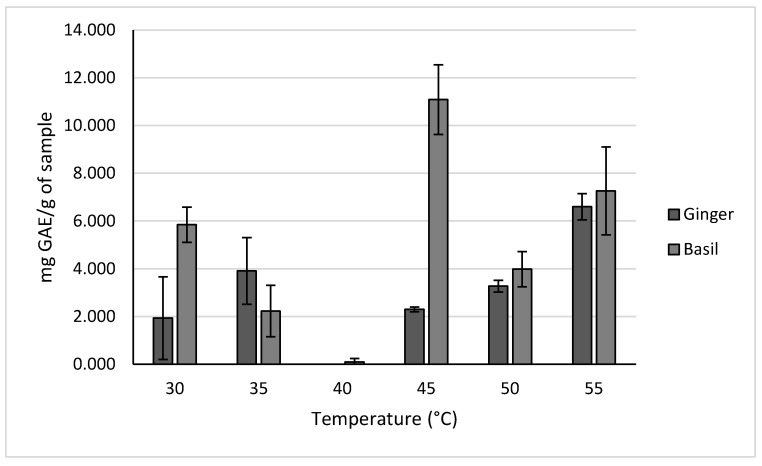
Polyphenol concentrations for ginger and basil across different extraction temperatures.

The results for different extraction temperatures are shown in Figure 7. Temperatures ranging from 30 °C to 55 °C were used due to 30 °C being the lowest temperature on the instrument and 55 °C being the highest temperature at which the phytochemicals could be tested before risking degradation. Based on these results, it was determined that a temperature of 45 °C resulted in the highest yield for holy basil at 11.086 ± 1.463 mg GAE/g of sample. Meanwhile, a temperature of 55 °C resulted in the highest yield for ginger at 7.264 ± 1.840 mg GAE/g of sample. It was observed that, at certain temperatures, the concentration of polyphenols in the samples was lower than the limit of detection.

A temperature of 45 °C was used to test the holy basil samples due it to resulting in the highest yields during the temperature trials, while a temperature of 35 °C was used to test the ginger samples due to it resulting in similar yields to reflux extraction.

**Figure 8:**
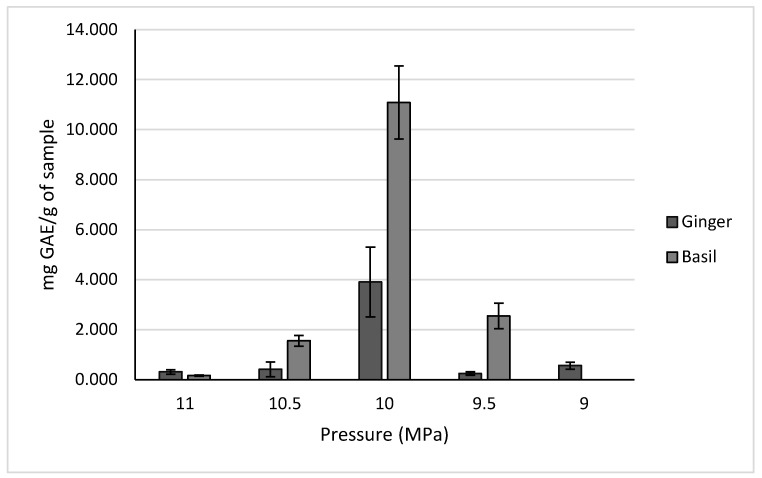
Polyphenol concentrations for ginger and basil across different extraction pressures.

The results for the different pressure trials are shown in Figure 8. For both ginger and holy basil, a pressure of 10 MPa resulted in the highest yields at 7.264 ± 1.840 mg GAE/g of sample and 11.086 ± 1.463 mg GAE/g of sample, respectively.

#### 2.2.2. Anthocyanins of Aronia

**Figure 9:**
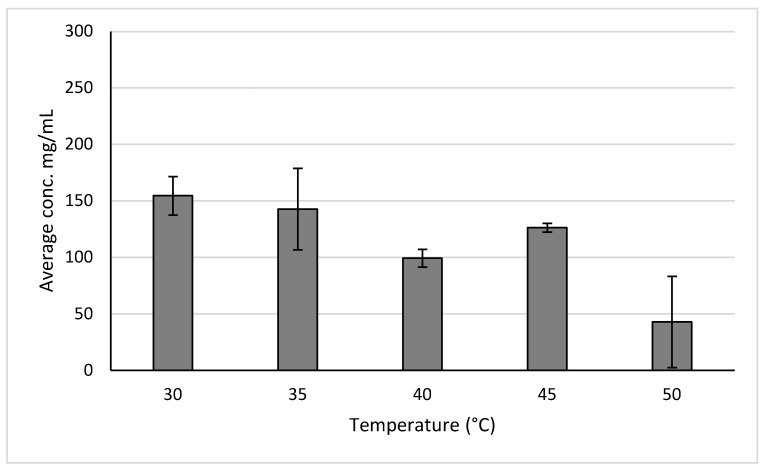
Anthocyanin concentrations across different extraction temperatures for aronia berries.

The results show that, when measuring anthocyanins after the temperature test, the best performing temperature was 30 degrees, resulting in a yield of 154.5 ± 17.1 mg/mL (Figure 9).

**Figure 10:**
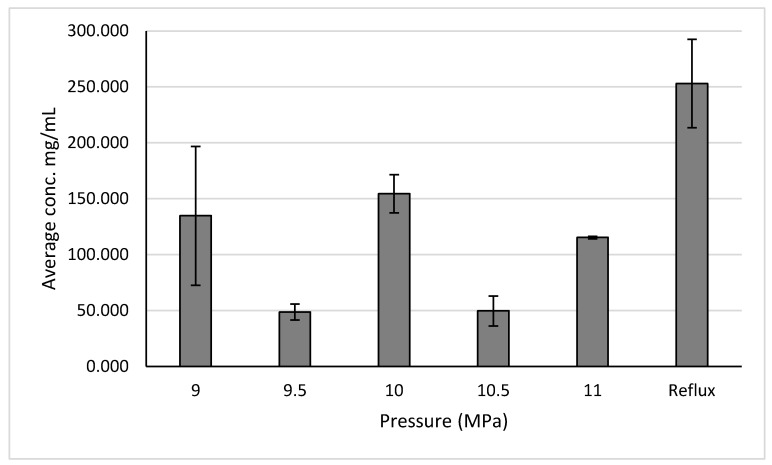
Anthocyanin concentrations across different extraction pressures for aronia berries.

A temperature of 30 °C was found to produce the highest yield when extracting across temperatures. The results show that, when measuring anthocyanins after the pressure test, the best performing pressure was 10 MPa, as it resulted in a yield of 154.5 ± 17.1 mg/mL (Figure 10).

#### 2.2.3. Optimal Extraction Conditions and Comparison to Literature Data

The optimal high-pressure extraction temperatures differed between crops, though all optimal extractions were performed using a pressure of 10 MPa. A comparison of the yields produced under optimal high-pressure extraction to those produced under reflux extraction and the literature data can be seen in Table 2. Most notably, for holy basil and aronia, the parameters that produced the highest yields were also the parameters that produced yields most similar to reflux extraction. Meanwhile, for ginger, the extraction temperature that produced similar yields to reflux extraction, which was 50 °C, was different to that that produced the highest yields, which was 55 °C.

Additional raw data in support of all results are available in the Supplemental Materials.

## 3. Materials and Methods

### 3.1. Sample Sourcing and Processing

Ginger samples were grown in a high tunnel at Randolph Farm at Virginia State University and harvested at six months of age. The samples were stored in vacuum-sealed bags at −20 °C no later than five hours after harvesting until analysis.

Holy basil samples were grown in Princess Anne, Maryland, at the University of Maryland Eastern Shore’s extension farm. The samples were grown in individual pots and harvested throughout the growing cycle. The holy basil was cut approximately one inch from the root throughout each harvest and then later separated into piles of flowers, leaves, and stems. The samples were vacuum-sealed and kept at −20 °C until it was time for analysis.

Aronia samples were grown at the University of Maryland’s Wye Research and Education Center in Queenstown, MD. Fruit samples were transported from the farm on ice and stored at −20 °C until it was time for analysis.

### 3.2. Automatic Steam Distillation Methods

To assess the optimal parameters for distilling samples using an automatic steam distiller, essential oil yields were determined gravimetrically.

For all steam distiller trials, 1 g of sample was first prepared and combined with 50 mL of distilled water. This sample was then subjected to steam distillation using a K-365 EasyDist automatic wet distillation apparatus [Buchi, Flawil, Switzerland], with the parameters used modified between trials.

After distillation, the resulting sample was combined with 50 mL of hexane and underwent liquid–liquid extraction. The fraction containing the hexanes and essential oils was then processed using a rotary evaporator, by which the hexanes were evaporated, leaving behind the essential oils. The mass of the essential oils was then determined gravimetrically and compared to the original mass of the sample to determine the mg of oil per g of sample using the following equation:

mg oil/g sample = final mass of oils∙1000/initial sample mass

#### 3.2.1. Steam Time Trials

For each steam time trial, the automatic distiller was set to operate at 90% steam power with varying amounts of steam time. For ginger, 150, 180, 210, 240, and 270 s steam times were tested. For holy basil and aronia, 180, 210, 240, 270, and 300 s steam times were tested.

#### 3.2.2. % Steam Power Trials

For ginger and holy basil % steam power trials, the steam time was set to 210 s, while 240s was used for aronia. The automatic distiller was then set to use 80%, 85%, 90%, 95%, and 100% steam power for all three crops.

### 3.3. Traditional Extraction Methods

For traditional extraction methods, 5 g of each sample was obtained. The sample was then added to 50 mL of 50% ethanol in water and refluxed for 48 h. After this, the sample was vacuum-filtered and subjected to phytochemical screening.

### 3.4. Pressure Extractor Method

Before each pressure extractor trial, a leak test was performed on the instrument. The parameters were first optimized and then used as follows: temperature—100 °C; pressure—10 MPa; solvent—ethanol/water 50/50% vol; hold time—4 min; extraction cell—all volumes.

**Figure 11:**
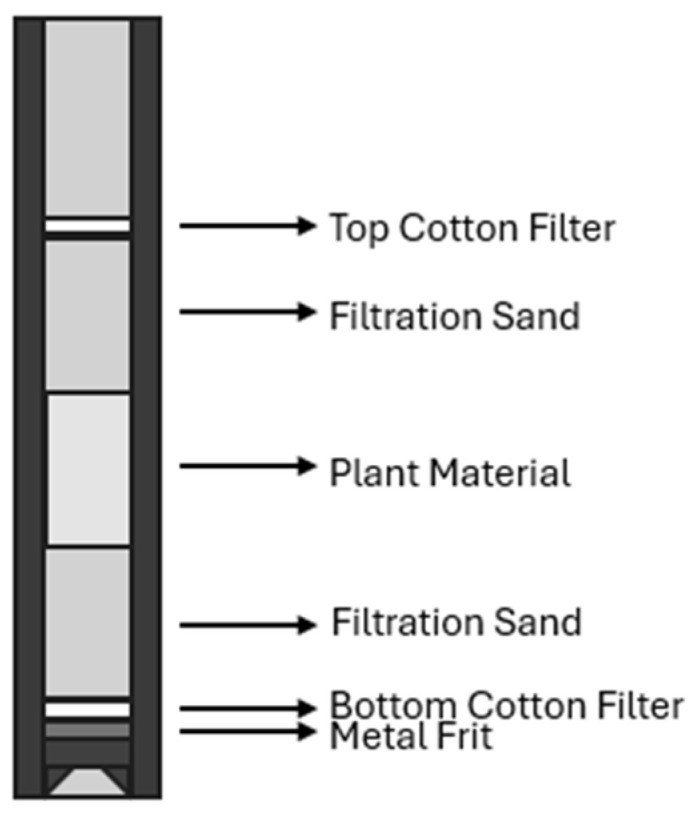
Packing scheme used for extraction cells.

Once a leak test was completed, 2 g of sample was prepared, alongside 20 g of fine-quality sand. First, a small cotton filter was placed on top of the metal frit in a cell. Once the cell was completely sealed on the bottom, half of the sand was filtered into the cell, followed by the whole sample and then the final half of the sand (Figure 11). The cell was then covered with a larger cotton filter provided by the company, and the samples were then placed into a SpeedExtractor E-916 [Buchi, Flawil, Switzerland] for extraction.

The extraction temperature and pressure were tested for all samples. All other parameters were held constant as follows: cell—20 mL; solvent—ethanol/water 50/50%; # of cycles—1; flush with solvent—0.5 min; flush with gas—2 min; hold time—15 min; heat-up time—1 min; discharge time—2 min.

#### 3.4.1. Temperature Trials

The first parameter tested was the extraction temperature. For each type of sample, extraction temperatures of 30 °C, 35 °C, 40 °C, 45 °C, and 50 °C were tested for all crops, with 55 °C also being tested for ginger and basil. Additionally, a pressure of 10 MPa was used for all trials.

#### 3.4.2. Pressure Trials

The next parameter tested was extraction pressure. The pressures tested were 9, 9.5, 10, 10.5, and 11 MPa for all crops. Temperatures of 35 °C, 45 °C, and 30 °C were used for ginger, holy basil, and aronia, respectively.

### 3.5. Phytochemical Screening

#### 3.5.1. Polyphenols

**Figure 12:**
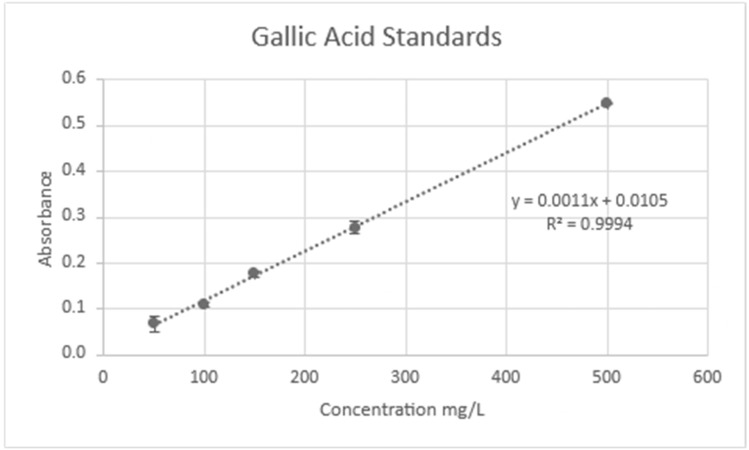
Calibration curve: polyphenols.

The polyphenol content was measured using a method outlined by Nowak et al. [37]. A calibration curve was created with a gallic acid standard (Figure 12). Then, 20 microliters of sample, 1580 microliters of distilled water, and 100 microliters of Follins Reagent were combined. The samples were incubated at room temperature for five minutes. After incubation, 300 microliters of 20% *w*/*v* sodium carbonate was added. The samples were then incubated again at 40 °C. The absorbance was then measured at 765 nm, and the total polyphenol concentration (TPC) in mg of gallic acid equivalents (GAE)/g of Dry Weight (DW) or Fresh Weight (FW) was then determined using the following equation:

TPC = (A × V)/(1.1 × 1000 × m) × DF

where A = the average absorbance, V = the collected volume of extract, m = the mass of the sample used for extraction, and DF = the dilution factor.

**Figure 13:**
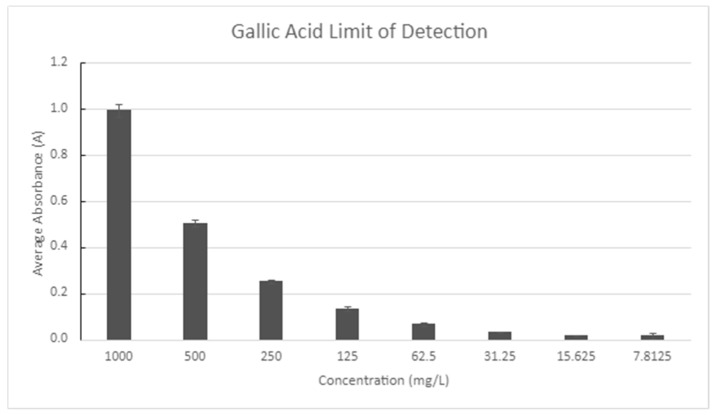
Limit of detection: polyphenols.

A test to determine the limit of detection of the polyphenol test was performed using known standards of gallic acid (Figure 13). The standards were tested until the absorbance reached zero and a non-linear relationship was found with the concentration.

#### 3.5.2. Anthocyanins

The anthocyanin concentration was calculated through a method developed by Giusti and Wrolstad [38]. Two sets of samples were created using two separate pH buffers: one sample using 0.025 M potassium chloride, which was adjusted to pH 1 using HCL, and one sample using 0.4 M sodium acetate solution at pH 4.5. Absorbance was measured at 520 and 700 nm using a Cary60 [Agilent, Santa Clara, CA, USA] spectrophotometer blanked with distilled water. The following formula was used to calculate the absorbance (A):

A = (A_520_ − A_700_) pH_1.0_ − (A_520_ − A_700_) pH_4.5_A = (A_520_ − A700) pH_1.0_ − (A_520_ − A_700_) pH_4.5_.

The total anthocyanin concentration per sample was calculated using the following formula:

Anthocyanin content (mg/L) = (A × MW × DF × 1000)/(ε × 1)

where MW is the molecular weight of cyanidin-3-O-glucoside (449.2 g/mol), DF is the dilution factor, and ε is the molar extinction coefficient of cyanidin-3-glucoside (ε = 26,900 L cm^−1^ mol^−1^). The anthocyanin content is calculated in cyanidin-3-glucoside equivalents in mg/L.

**Figure 14:**
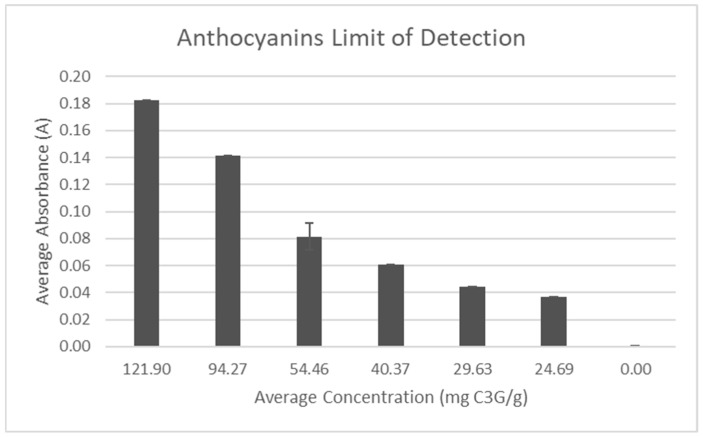
Anthocyanin limit of detection test.

A test to determine the limit of detection of the anthocyanin test was performed using dilutions of pure *Aronia* juice mixed with DI water (Figure 14). The dilutions were tested until the absorbance reached zero and a non-linear relationship was found with the concentration.

## 4. Conclusions

In this manuscript, we developed comparable modern methods of high-pressure extraction and automatic distillation. Due to the lower concentrations produced by high-pressure extraction for holy basil and aronia, the procedures should be further examined.

The goal of this study was to develop procedures that result in concentrations of extracts/yields comparable to those obtained for almost a century using traditional extraction methods, as opposed to the goal of maximizing the yield, which is a goal relevant for technology transfer but less relevant for horticultural phytochemistry research. As a result, this research has some limitations related to the experimental design choice. Separate research could be conducted to maximize the yield.

The highest yield of essential oils for ginger was 61.52 ± 0.61 mg/g of sample when 90% steam power was used for 210 s. The highest yield of essential oils for basil was 55.81 ± 1.97 mg/g of sample when 90% steam power was used for 210 s. The highest yield of essential oils for aronia was 45.79 ± 1.38 mg/g of sample when 90% steam power was used for 240 s. The highest concentration of polyphenols in ginger was 7.26 ± 1.84 mg GAE/g when using 10 MPa at 55 °C. The highest concentration of polyphenols in basil, as well as the closest to the reflux extraction concentration, was 11.09 ± 1.46 mg GAE/g when using 10 MPa at 45 °C. The highest concentration of anthocyanins in aronia, as well as the closest to the reflux extraction concentration, was 154.50 ± 17.10 mg/mL when using 10 MPa at 30 °C.

## Figures and Tables

**Table 1:** Data obtained from the literature for essential oil concentrations of holy basil and ginger in comparison with those obtained using the developed methods.

Crop	Place of Growth	Oil Concentration	Conditions	Reference
Holy Basil	Maryland, USA	55.81 ± 1.97 mg/g of sample	Automatic steam distillation 90% steam power 210 s steam time	
Holy Basil	Wyoming, USA	0.68 ± 0.05 g oil/100 g leaves	60 min of steam distillation	[19]
Holy Basil	Georiga (lat. 33°53′55.5″ N; long. 83°22′09.2″ W), USA	0.65 (*w*/*w*)	Hydrodistillation	[23]
Holy Basil	Australia	4.1% *w*/*v*	Hydrodistillation	[24]
Holy Basil	Himachal Pradesh, India	2.74 ± 0.57% *w*/*v*	3 h hydrodistillation	[25]
Ginger	Virginia, USA	61.52 ± 0.61 mg/g of sample	Automatic steam distillation 90% steam power 210 s steam time	
Ginger	Zaria, Nigeria	2.4%	6 h distillation	[26]
Ginger	Assan, India	4.17 ± 0.05%	Hydrodistillation	[27]
Ginger	Nottingham, UK	0.35% (*w*/*w*)	Microwave-assisted hydrodistillation	[28]
Ginger	Sichuan Province, China	2.5%	Microwave-assisted hydrodistillation	[29]

**Table 2:** Literature data for polyphenol concentrations in holy basil and ginger and for anthocyanins in aronia.

Crop	Place of Growth	Phytochemical Concentration	Conditions	Reference
Holy Basil	Maryland, USA	11.086 ± 1.463 mg GAE/g of sample	High-pressure extraction 45 °C, 10 MPa	
Holy Basil	Maryland, USA	32.709 ± 5.222 mg GAE/g of sample	48 h reflux in 50% ethanol at 30 °C	
Holy Basil	Alabama	31.37 ± 1.29	80% methanol and a dry sample	[30]
Holy Basil	Nakhon Pathom, Thailand	23.19 mg GAE/gDW	25 °C absolute methanol, 3 h	[31]
Holy Basil	India	2.18 ± 0.015 mg/mL GAE	Overnight solid–liquid extraction by emersion in methanol	[32]
Ginger	Virginia, USA	6.600 ± 0.549 mg GAE/g of sample	High-pressure extraction 55 °C, 10 MPa	
Ginger	Virginia, USA	3.273 ± 0.248 mg GAE/g of sample	High-pressure extraction 50 °C, 10 MPa	
Ginger	Virginia, USA	3.341 ± 2.066 mg GAE/g of sample	48 h reflux in 50% ethanol at 30 °C	
Ginger	Morocco	322.11 µg GAE/mg Ext	40 °C ethanol extraction	[33]
Ginger	Riyadh, Saudi Arabia	2.4 mg GAE/g	25 °C methanol extraction	[34]
Ginger	Malaysia	2.63 mg GAE/g	ethanol maceration	[35]
Aronia	Maryland, USA	154.5 ± 17.1 mg of anthocyanins/mL	High-pressure extraction 30 °C, 10 MPa	
Aronia	Maryland, USA	253.0 ± 39.56 mg of anthocyanins/mL	48 h reflux in 50% ethanol at 30 °C	
Aronia	Connecticut, USA	0.469 ± 0.008 mg/g Cy3Glu 9.00 ± 0.05 mg/g Cy3Gal	2 g of berry powder was diluted in 40 mL of 70% acetone, 29.5% water, and 0.5% acetic acid; sonicated for 5 min; and centrifuged at 950 g for 10 min	[21]
Aronia	Finland	38% Cyanidin-3-galactoside	Extracted with 4% acetic acid in 65% aqueous methanol	[36]

## Data Availability

All data are available in this paper or in Supplemental Materials.

## Supplementary Materials

The following supporting information can be downloaded at: https://www.mdpi.com/article/10.3390/molecules30102199/s1, Table S1: The milligram of essential oil per gram of ginger across different steam times; Table S2: The milligram of essential oil per gram of holy basil across different steam times; Table S3: The milligram of essential oil per gram of aronia across different steam times; Table S4: The milligram of essential oil per gram of ginger across different % steam power values; Table S5: The milligram of essential oil per gram of holy basil across different % steam power values; Table S6: The milligram of essential oil per gram of aronia across different % steam power values; Table S7: Ginger polyphenols across different extraction temperatures; Table S8: Holy basil polyphenols across different extraction temperatures; Table S9: Ginger polyphenols across different extraction pressures; Table S10: Holy basil polyphenols across different extraction pressures; Table S11: Aronia anthocyanins across different extraction temperatures; Table S12: Aronia anthocyanins across different extraction temperatures.

## Abbreviations

The following abbreviations are used in this manuscript:

UV/Vis	ultraviolet–visible spectrophotometry
nm	Nanometer
ml	Milliliter
μL	microliter
M	molar
g	gram
mg	milligram
MPa	Megapascal
DF	dilution factor
DI water	de-ionized water
°C	degree Celsius
GCMS	gas chromatography–mass spectroscopy
C3G	cyanidin-3-glucoside
DW	Dry Weight
FW	Fresh Weight
GAE	gallic acid equivalents
QE	Quercetin Equivalents
