# Supplementary material for: Development of High-Pressure Extraction and Automatic Steam Distillation Methods for Aronia mitschurinii, Juvenile Ginger, and Holy Basil Plants
Source: Molecules. 2025 May 17;30(10):2199. doi: 10.3390/molecules30102199 (PMC12113872; doi:10.3390/molecules30102199)
Supplement: Supplementary file 1 [file molecules-30-02199-s001.zip › molecules-3598505-supplementary.pdf]

Development methods for using High-Pressure Extractors Extraction and Automatic Steam Distillers Distillation for *Aronia mitschurinii*, Juvenile ginger, and Holy Basil plants.

Sara Lahoff [1], Ezra Cable [1], Ryan More[1], and Victoria Volkis[1,*]

[1] Department of Natural Sciences, The University of Maryland Eastern Shore, 3, Princess Anne, Maryland 21853, USA
* Correspondence: vvollkis@umes.edu

Table S1. mg of essential oil per gram of ginger samples across different steam times. A steam power of 90% was used for all trials.

**Steam Time (s)**	**150**	**180**	**210**	**240**	**270**
Trial 1	41.58	51.66	61.37	49.95	4.335
Trial 2	47.76	50.68	62.19	48.19	4.545
Trial 3	44.04	48.99	61.00	47.47	4.266
Average	44.46	50.44	61.52	48.54	4.382
Standard Deviation	3.110	1.3487	0.6101	1.273	0.1456

Table S2. mg of essential oil per gram of holy basil samples of essential oil from holy basil across different steam times. A steam power of 90% was used for all trials.

**Steam Time (s)**	**180**	**210**	**240**	**270**	**300**
Trial 1	42.45	54.05	52.99	47.15	4.0354
Trial 2	43.13	55.44	48.39	45.69	3.4034
Trial 3	46.12	57.94	47.01	50.52	3.4014
Average	43.90	55.81	49.46	47.78	3.613
Standard Deviation	1.955	1.970	3.132	2.476	0.3655

Table S3. mg of essential oil per gram of aronia samples across different steam times. A steam power of 90% was used for all trials.

**Steam Time (s)**	**180**	**210**	**240**	**270**	**300**
Trial 1	39.25	43.87	47.28	43.69	40.00
Trial 2	35.68	42.96	44.55	38.95	38.35
Trial 3	38.42	39.68	45.54	41.38	43.91
Average	37.79	42.17	45.79	41.34	40.75
Standard Deviation	1.872	2.201	1.382	2.373	2.858

Table S4. mg of essential oil per gram of ginger samples across different % steam power. A steam time of 210 s was used for all trials.

% steam power	80	85	90	95	100
Trial 1	37.15	45.10	61.37	55.94	48.39
Trial 2	38.46	48.24	62.19	51.18	49.51
Trial 3	42.45	42.83	61.00	51.36	52.16
Average	39.35	45.39	61.52	52.83	50.02
Standard Deviation	2.761	2.718	0.610	2.700	1.935

Table S5. mg of essential oil per gram of holy basil samples across different % steam power. A steam time of 210 s was used for all trials.

% steam power	80	85	90	95	100
Trial 1	40.31	46.21	54.05	41.67	46.00
Trial 2	38.42	44.27	55.44	40.47	48.14
Trial 3	43.31	41.62	57.94	41.41	46.67
Average	40.68	44.03	55.81	41.18	46.94
Standard Deviation	2.463	2.304	1.970	0.629	1.097

Table S6. mg of essential oil per gram of aronia samples across different % steam power. A steam time of 210 s was used for all trials.

% steam power	80	85	90	95	100
Trial 1	33.46	38.54	47.28	42.62	23.09
Trial 2	32.55	36.89	44.55	44.60	24.19
Trial 3	35.40	41.08	45.54	44.47	29.10
Average	33.805	38.836	45.794	43.894	25.461
Standard Deviation	1.453	2.112	1.382	1.108	3.197

Table S7. Ginger polyphenols across different extraction temperatures. A pressure of 10 MPa was used for all trials.

Temperature (°C)	30	35	40	45	50	55	Reflux
Abs. 1	0.029	0.031	0.000	0.027	0.033	0.055	0.042
Abs. 2	0.000	0.051	0.000	0.028	0.037	0.058	0.045
Abs. 3	0.035	0.035	0.000	0.027	0.034	0.063	0.018
Average Abs.	0.021	0.039	0.000	0.027	0.024	0.059	0.035
Abs. Deviation	0.019	0.011	0.000	0.027	0.023	0.004	0.015
Conc. 1	2.468	2.85	0	2.291	3.123	6.123	4.309
Conc. 2	0	5.495	0	2.400	3.559	6.477	4.745
Conc. 3	3.341	3.395	0	2.209	3.136	7.200	0.968
Average conc. mg GAE/g of sample	1.936	3.914	0.000	2.300	3.273	6.600	3.341

Conc. Deviation	1.733	1.397	0.000	0.096	0.248	0.549	2.066

Table S8. Holy basil polyphenols across different extraction temperatures. A pressure of 10 MPa was used for all trials.

Temperature (°C)	30	35	40	45	50	55	Reflux
Abs. 1	0.053	0.019	0.013	0.104	0.034	0.053	0.208
Abs. 2	0.059	0.035	0.000	0.087	0.042	0.059	0.283
Abs. 3	0.048	0.026	0.000	0.084	0.044	0.079	0.259
Average Abs.	0.054	0.027	0.004	0.092	0.040	0.064	0.250
Abs. Deviation	0.006	0.008	0.008	0.011	0.005	0.014	0.038
Conc. 1	5.795	1.200	0.273	12.764	3.136	5.836	26.986
Conc. 2	6.614	3.355	0.000	10.418	4.309	6.614	37.214
Conc. 3	5.141	2.141	0.000	10.077	4.500	9.341	33.927
Average conc. mg GAE/g of sample	5.850	2.232	0.091	11.086	3.982	7.264	32.709
Conc. Deviation	0.738	1.080	0.157	1.463	0.738	1.840	5.222

Table S9. Ginger polyphenols across different extraction pressures. Temperatures of 40°C were used for all trials.

Pressure (MPa)	11	10.5	10	9.5	9	Reflux
Abs. 1	0.029	0.058	0.031	0.025	0.041	0.042
Abs. 2	0.037	0.025	0.051	0.023	0.057	0.045
Abs. 3	0.026	0.026	0.035	0.032	0.042	0.018
Average Abs.	0.031	0.037	0.039	0.026	0.046	0.035
Abs. Deviation	0.006	0.019	0.011	0.005	0.009	0.015
Conc. 1	0.293	0.757	2.850	0.230	0.479	4.309
Conc. 2	0.414	0.235	5.495	0.192	0.726	4.745
Conc. 3	0.240	0.250	3.395	0.331	0.490	0.968
Average conc. mg GAE/g of sample	0.316	0.414	3.914	0.251	0.565	3.341
Conc. Deviation	0.089	0.297	1.397	0.072	0.140	2.066

Table S10. Holy basil polyphenols across different extraction pressures. Temperatures of 40°C were used for all trials

Pressure (MPa)	11	10.5	10	9.5	9	Reflux
Abs. 1	0.020	0.122	0.104	0.190	0.000	0.208
Abs. 2	0.023	0.108	0.087	0.192	0.000	0.283
Abs. 3	0.020	0.095	0.084	0.135	0.000	0.259
Average Abs.	0.021	0.109	0.092	0.172	0.000	0.25
Abs. Deviation	0.001	0.013	0.011	0.032	0.000	0.038
Conc. 1	0.156	1.779	12.764	2.837	0.000	26.986
Conc. 2	0.191	1.550	10.418	2.861	0.000	37.214

Conc. 3	0.154	1.351	10.077	1.962	0.000	33.927
Average conc. mg GAE/g of sample	0.167	1.560	11.086	2.554	0.000	32.709
Conc. Deviation	0.021	0.214	1.463	0.512	0.000	5.221

Table S11. Aronia anthocyanin concentrations across different temperatures. Pressures of 10 MPa were used for all trials.

Temperature (°C)	30	35	40	45	50	Reflux
Stand. Abs. 1	0.0417	0.0313	0.0310	0.0393	0.0296	0.0831
Stand. Abs. 2	0.0518	0.0441	0.0270	0.0364	0.0266	0.0821
Stand. Abs. 3	0.0453	0.0528	0.0311	0.0377	0.0386	0.0621
Average Stand. Abs.	0.046	0.043	0.030	0.038	0.029	0.076
Abs. Deviation	0.0051	0.0108	0.0023	0.0015	0.0092	0.0118
Conc. 1	139.3	104.5	103.5	131.3	13.76	277.5
Conc. 2	173.0	147.3	90.17	121.6	88.84	274.2
Conc. 3	151.3	176.3	103.9	125.9	25.78	207.4
Average conc. mg /mL	154.5	142.7	99.19	126.2	42.79	253.0
Conc. Deviation	17.10	36.12	7.811	3.851	40.33	39.56

Table S12. Aronia anthocyanin concentrations across different pressures. Temperatures of 30°C were used for all trials.

Pressure (MPa)	11	10.5	10	9.5	9	Reflux
Stand. Abs. 1	0.0078	0.0000	0.0417	0.0108	0.0018	0.0831
Stand. Abs. 2	0.0138	0.0124	0.0518	0.0040	0.0000	0.0821
Stand. Abs. 3	0.0168	0.0000	0.0453	0.0121	0.0000	0.0621
Average Stand. Abs.	0.013	0.004	0.046	0.009	0.001	0.076
Abs. Deviation	0.0046	0.0072	0.0051	0.0044	0.0010	0.0118
Conc. 1	26.05	0.000	139.3	36.07	1.202	277.5
Conc. 2	46.09	41.41	173.0	13.36	0.000	274.2
Conc. 3	56.11	0.000	151.3	40.41	0.000	207.4
Average conc. mg /mL	42.75	13.80	154.5	29.95	0.4008	253.0
Conc. Deviation	15.30	23.91	17.10	14.53	0.6942	39.56
